# Role of nutraceuticals during the transition period of dairy cows: a review

**DOI:** 10.1186/s40104-020-00501-x

**Published:** 2020-08-27

**Authors:** Vincenzo Lopreiato, Matteo Mezzetti, Luca Cattaneo, Giulia Ferronato, Andrea Minuti, Erminio Trevisi

**Affiliations:** 1 Department of Animal Sciences, Food and Nutrition, Faculty of Agriculture, Food and Environmental Science, Università Cattolica del Sacro Cuore, 29122 Piacenza, Italy; 2 PRONUTRIGEN-Centro di Ricerca Nutrigenomica e Proteomica, Università Cattolica del Sacro Cuore, Piacenza, Italy

**Keywords:** Dairy cow, Essential fatty acid, Immunometabolism, Methyl donor, Nutraceuticals, Nutrition, Peripartum, Phytoproduct, Yeast culture

## Abstract

The transition period of dairy cattle is characterized by a number of metabolic, endocrine, physiologic, and immune adaptations, including the occurrence of negative energy balance, hypocalcemia, liver dysfunction, overt systemic inflammatory response, and oxidative stress status. The degree and length of time during which these systems remain out of balance could render cows more susceptible to disease, poor reproductive outcomes, and less efficient for milk production and quality. Studies on both monogastrics and ruminants have reported the health benefits of nutraceuticals (e.g. probiotics, prebiotics, dietary lipids, functional peptides, phytoextracts) beyond nutritional value, interacting at different levels of the animal’s physiology. From a physiological standpoint, it seems unrealistic to disregard any systemic inflammatory processes. However, an alternate approach is to modulate the inflammatory process per se and to resolve the systemic response as quickly as possible.

To this aim, a growing body of literature underscores the efficacy of nutraceuticals (active compounds) during the critical phase of the transition period. Supplementation of essential fatty acids throughout a 2-month period (i.e. a month before and a month after calving) successfully attenuates the inflammatory status with a quicker resolution of phenomenon. In this context, the inflammatory and immune response scenario has been recognized to be targeted by the beneficial effect of methyl donors, such as methionine and choline, directly and indirectly modulating such response with the increase of antioxidants GSH and taurine. Indirectly by the establishment of a healthy gastrointestinal tract, yeast and yeast-based products showed to modulate the immune response, mitigating negative effects associated with parturition stress and consequent disorders.

The use of phytoproducts has garnered high interest because of their wide range of actions on multiple tissue targets encompassing a series of antimicrobial, antiviral, antioxidant, immune-stimulating, rumen fermentation, and microbial modulation effects. In this review, we provide perspectives on investigations of regulating the immune responses and metabolism using several nutraceuticals in the periparturient cow.

## Background

Around parturition, dairy cows experience the majority of health problems as a consequence of improper adaptations from a non-productive period (dry) to the onset of a new lactation. Thus, the purpose of many researches, throughout the second half of the XX century and current day, has been focused on the influence of the “transition period” (TP), to date established to start at the beginning of dry-off, on health and immune function, the interplay between the endocrine and immune systems, and, more recently, nutrition linked to immune function.

**Fig. 1:**
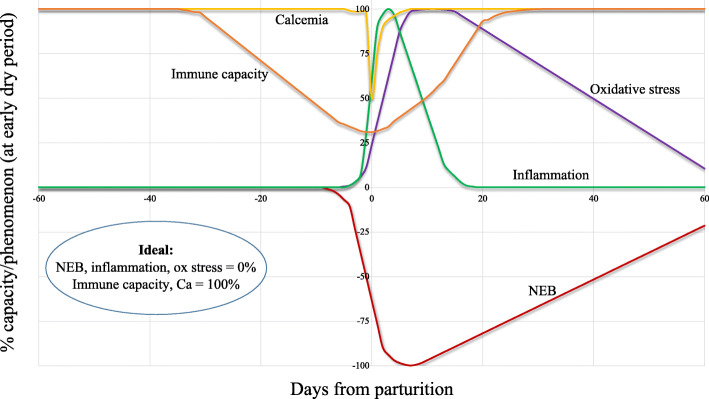
Theoretical pattern of changes in the main physiological aspects of healthy subjects during the transition period. Ideally, the Negative energy balance (NEB), inflammation, and oxidative stress would be close to zero (i.e. absence of the phenomena), whereas the immunocompetence and the calcemia would be close to 100% of their optimal level [2]

To a large extent, the health problems during the periparturient period relate to cows having difficulty in adapting to the nutrient needs for lactation [1]. This may result in physiological imbalance (Fig. 1), a situation where the regulatory mechanisms are insufficient for the animals to function optimally leading to a high risk of a complex of digestive, metabolic [3], and infectious problems [2]. The risk of infectious diseases increases if the immune functions (such as phagocytosis, oxidative burst, chemotaxis, cell-cell interaction) are impaired. Nutrition plays a pivotal role in immune response, and the effect of nutrition may occur directly through nutrients or indirectly through biological active metabolites, for example, in situations with physiological imbalance [1].

Use of nutraceuticals has received increasing attention for the improvement of animal health, welfare, and productivity in herd health management. Studies on nutraceuticals investigated their use as nutrients, dietary supplements, herbal products, and processed feeds (including dietary fiber, probiotics and prebiotics, polyunsaturated fatty acids, antioxidant vitamins and trace minerals, and phytoactive compounds). Results revealed their potential to support the immune system and metabolic activity of the main organs (such as liver, mammary gland, and gut) prior to and after parturition, especially when inflammatory response activate various components of the immune system and alterations in metabolism [4]. These motives have promoted the value of nutraceuticals, where complex multitarget poly-pharmacological mechanisms, such as activation of antioxidant defense and anti-inflammatory pathways (along with beneficial effects on cells through integrity, survival, proliferation, and differentiation), are exhibited [5].

Considering the present framework, this review aims to summarize findings and potential perspectives of those nutraceutical compounds that are not practically considered in the diet requirement formulations for high-yield dairy cows, especially during the transition period. We also know the relevant importance of certain vitamins and minerals functioning as nutraceuticals to modulate the oxidative stress response. However, in this review, we do not specifically discuss their use and outcomes since several scientific impacting and influential reviews have been already published [6].

### The transition cow – an overview

**Fig. 2:**
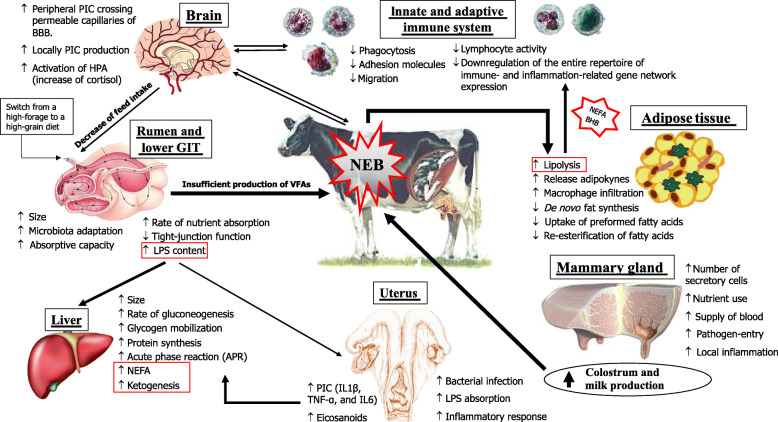
Peripartal adaptations of the key tissue such as liver, mammary gland, adipose, rumen, uterus, brain, as well as the immune system. It is highlighted the main factors that influence the functional response capacity of the key tissues involved in the homeorhetic adaptation during the transition period. NEB: negative energy balance; GIT: gastro-intestinal tract; BHB: β-hydroxybutyrate; NEFA: Non-esterified fatty acids; LPS: lipopolysaccharide; HPA: hypothalamus-pituitary-adrenal axis; PIC: pro-inflammatory cytokines; BBB: blood-brain barrier

In one of his influential review articles published in 1999, Prof. J. Drackley [7] argued that the biology underlying the transition to lactation was the “final frontier” in our understanding of the dairy cow. Since then, a number of relevant in-depth studies have uncovered most of the “obscured field” of the transition period. Such researches have demonstrated that immune cells are directly involved in a surprising array of metabolic functions, including the maintenance of gastrointestinal function, control of adipose tissue lipolysis, which in turn determines the liver functionality, and regulation of insulin sensitivity in multiple tissues [8]. On the other hand, it was also postulated and highlighted that metabolic changes related to energy and calcium supply in support of lactation, occurring concurrently, impair the innate immune response [9, 10] (Fig. 1). Clearly, the mechanisms linking these changes and metabolic challenges during the transition period (Fig. 2) are only partially understood, further demanding the question: what is the trigger of the metabolism and immune imbalance in the peripartum?

During TP and mainly after parturition, circulating glucose is prioritized to the non-insulin-dependent glucose transporters, which are only expressed on immune cells and the mammary gland. Nevertheless, the massive glucose requirements of an activated immune system during systemic inflammation could further reduce the energy available for the mammary gland, aggravating the negative energy balance (NEB) occurring in early lactation [11]. Data obtained in Holstein lactating cows after a stimulation with LPS indicated that an acutely activated immune system uses > 1 kg of glucose within 720 min from the onset of inflammation [11]. When NEB occurs, mobilization of body fats and proteins are induced, and non-esterified fatty acids (NEFA) and amino acids are used as fuel sources by the liver [12]. However, a severe NEB occurring in TP could induce a NEFA overload in the liver, increasing the release of beta hydroxybutyrate (BHB) in blood and impairing pivotal functions [7].

Oxidative stress also occurs during this period and is driven by the imbalance between the production of reactive oxygen metabolites (ROM), reactive nitrogen species (RNS), and the neutralizing capacity of antioxidant mechanisms in tissues and blood. The increase in oxidative stress and inflammation during this period is also associated with a reduction in liver functionality, for which measurement of APP can provide a useful tool to assess liver function as well as inflammation [13]. In this context, it is particularly important to understand how inflammatory processes in the peripheral tissues of transition cows signal their anorexic action to the hypothalamus [14]. Indeed, the hypothalamus is one of the key regions of the brain regulating energy balance as it receives and integrates input signals from the periphery, sensing humoral substances (such as nutrient-related metabolites, hormones and cytokines), but also integrating neural signals from other brain regions, the tongue or oronasal origin to adjust feed intake and energy expenditure [15] (Fig. 2).

Herein, we would like to point out the scenario occurring in the rumen during the transition period. Few studies have investigated the molecular adaptations of ruminal epithelium during the peripartum period [16]. These studies revealed the existence of interactions among genes of the immune system and those involved in the preparation for the onset of lactation, as well as the presence of growth factors that seem to be regulated after parturition [17]. The connections among ruminal fermentation, the ensuing ruminal epithelium adaptations, and the consequent system responses (Fig. 2) of the cow remain unclear. However, whether microbial metabolism could affect epithelial gene expression via metabolites remains uncertain. Also, the interaction of rumen epithelium with systemic immune response opens a new scenario in the management of forestomaches. The role of diet appears to be crucial (e.g. fermentability of carbohydrates, protein degradability) for nutrient balance and/or for microbiota composition, which might alter epithelium functioning (e.g. increase its permeability). Thus, feed molecules that favor rumen stability should be studied to discover their effects on modulation of the rumen fermentation, microbiota biodiversity, and protection of epithelial cells.

### Essential fatty acids

Mammals are able to synthetize all fatty acids that are essential for normal physiologic functions, except for polyunsaturated fatty acids (PUFA) ascribed in the n-3 and n-6 family, or the so-called “essential fatty acids,” as they must be provided through the diet. In TP, the amounts of PUFA decrease substantially in all the body compartments as compared with mid-lactation cows [18, 19], while the proportion of several saturated fatty acids (SFAs) increases [7, 20]. The primary and major source of n-3 fatty acids in ruminants is forage, particularly for grazing cattle, since forage galactolipids are rich in α-linolenic acid (ALA; C18:3n-3). Supplemental sources of n-3 PUFA in dairy cows diets include ALA from flaxseed as well as eicosapentaenoic (EPA; C20:5n-3) and docosahexaenoic acids (DHA; C22:6n-3) from fish oil [21]. Conversely, the n-6 PUFA are contained in many different feedstuffs (i.e. soybean, sunflower, corn, and cottonseeds), and their intake substantially increases around and after calving.

#### PUFAs

Supplementing PUFAs in dairy cows’ diets differentially affects productive performances depending on the dose and type administered (Table 1). Fish oil is known to decrease feed intake when supplemented in non-rumen protected forms and when percentages included in the diet are higher than 1% DM [21]. Decreased DMI reported with PUFAs could account for the reduced milk yield (MY) reported in several experiments. Furthermore, reduced butterfat has also been reported in several studies, administering PUFAs in different phases and suggesting incomplete protection against rumen biohydrogenation to intermediate isomers that are known to depress milk fat or are associated with milk fat depression (e.g. *trans*-10,*cis*-12 conjugated linoleic acid) [34]. Nevertheless, such results on productive performances are not consistent throughout the studies, indicating the pivotal role of diet formulation and lactation phase in affecting PUFAs’ effects on MY and composition.

**Table 1:** Summary of studies in periparturient dairy cows investigating the effects of supplemental essential fatty acids on performances

Nutraceutical^a^	Form	Dose^b^	Period	Effect^c^	Reference
ALA (C18:3n-3)	Whole flaxseed	9.7		Higher milk yields as compared with control cows or cows fed whole sunflower	[22]
		11	−49 to 28 DFC^d^	No effect on milk yield	[23]
				Reduced milk fat	[24]
				No effect on milk fat	[25]
	Extruded flaxseed	4 to 9.2	0 to 100 DFC	2.7% to 6.4% increase in MY	[26]
		4 to 15	Different phases	Reduced butterfat	[27, 28]
				No effect on milk fat	[29]
	Flaxseed oil			No effect on milk yield relative to palm oil	[30]
	Encapsulated flaxseed oil			Reduced milk fat as compared with cows supplemented with encapsulated saturated fatty acids	[31]
EPA (C20:5n-3) and DHA (C22:6n-3)	Fish oil	0.8 to 3		Reduced DMI; +/− MY: increase of MY adding up to 1% DM of fish oil, followed by a linear decrease with addition of fish oil up to 3% of DM; consistent decrease of milk fat content	[21, 32]
		2		No effect on milk yield	[33]
	Encapsulated fish oil	2.9	Transition period	No effect on milk yield and reduced butterfat as compared with cows fed encapsulated saturated fatty acids or flaxseed oil at the same rate	[31]

^a^ *ALA* Alpha-linolenic acid, *EPA* Eicosapentaenoic acid, *DHA* Docosahexaenoic acid

^b^ Expressed as % of dry matter whenever not differently indicated

^c^ *MY* milk yield

^d^ Days from calving

Supplementing rumen protected PUFAs during TP reduces the proportion of circulating SFAs, which markedly increase in the blood of early lactating cows due to the massive mobilization of NEFA from adipose tissue [20]. Such a shifted profile of circulating fatty acids is reflected in other body compartments. Increasing the amount of PUFAs in oocytes and follicular fluid has shown a positive effect on embryo implantation (EI), as excessive amounts of SFAs are known to impair oocyte competence and development [21]. A higher inclusion of PUFAs in white blood cells’ membranes at the expense of SFAs positively affect the immune functions [35]. In fact, excessive inclusion of SFAs resulting from adipose tissue mobilization in leukocytes membrane is known to play a role in triggering immune dysfunctions and unregulated inflammation in early lactation [36]. High amounts of SFAs modify proteins through fatty acylation, altering membrane fluidity, influencing how proteins anchor to the plasma membrane, and affecting the formation of glycoproteins that compose lipid rafts involved in lymphocytes activation, antibodies production, and inflammation [37].

Several SFAs (i.e. C12:0, C14:0 and C16:0) could also induce inflammation as they are similar to the acyl chains composing lipid A associated with bacterial lipopolysaccharides, which activate nuclear factor-κβ (NF-κB) mediated gene expression and increase inflammation and respiratory burst activity [19, 38]. Supplementing essential PUFAs (as rumen protected mainly) also exerts a direct effect on immune cells through modulating the expression of several transcription factors exerting pro- or anti-inflammatory actions. All n-3 PUFAs down-regulate the expression of adhesion molecules involved in inflammatory interactions between leukocytes and endothelial cells [39]. Linoleic acid (LLA; C18:2n-6), and particularly its conjugated isomers *cis*-9,*trans*-11 and *trans*-10,*cis*-12, interacts with peroxisome proliferation activated receptor (PPAR)-γ, while long chain n-3 (EPA and DHA) interacts with Toll-like receptors (TLRs)-2 and 4, PPARs, and sterol response element binding protein family [40, 41]. All these genes are involved in *NF-κB* regulation that orchestrates the production of pro-inflammatory cytokines in both immune and nonimmune cells [42]. The lack of n-3 and n-6 PUFAs in the post-partal period could, thus, induce uncontrolled inflammation.

#### n-6:n-3 ratio

The n-6:n-3 is the main indicator of PUFA, where values between 3.9 to 5.9 in dairy cows rations have been related to positive effects on immune functions and reproductive performances [43], while higher values are associated to the massive use of n-6 sources. Supplementing n-3 sources affects the composition of plasma, reducing the n-6:n-3 ratio to between − 3.7 to − 40.7 [26, 29]. Such a shift of fatty acids composition is reflected in many body compartments (i.e. cell membranes, follicular fluid, granulosa cells and oocytes), exerting different effects based on the dose, physical form, and administration time of n-3 rich feeds [21].

Decreased amount of arachidonic acid (AA; C20:4n-6) in cell membranes and follicular fluid induces anti-inflammatory and pro-resolving effects through shifting the oxylipids profile, class of lipid mediators produced by the enzymatic and non-enzymatic oxidation of PUFA [44], in favor of resolvins, protectins, lipoxins, and the 3 series prostaglandins and reducing the production of series 1 and 2 prostaglandins [45, 46]. This shifted oxylipid profile also improves the function of leukocytes (Table 2), as prostaglandin E_2_ is known to decrease the production of interferon gamma and proliferation of lymphocytes [65]. Altered oxylipid profile also improves the EI through ameliorating the development and maturation of oocytes [66, 67]. In fact, the series 1 and 2 prostaglandins (especially PGF2α released from the endometrium) are intimately involved in uterine involution and subsequent ovulation post-partum, negatively affecting the development of the morula and blastocyst stages [68]. Conversely, the 3 series prostaglandins improve the environment for embryo implantation and survival [64], and their production increases the lifespan of the corpus luteum, improves blastocyst cell numbers, and improves the maintenance of pregnancy [69]. Reduction in the concentration of linoleic acid (LA; C18:2n-6) in the follicular fluid also contributes to improving EI. In fact, AA is known to up-regulate the expression of steroidogenic acute regulatory protein, which mediates the transfer of cholesterol from the cytosol to the inner mitochondrial membrane, playing a pivotal role in steroid synthesis [70, 71].

**Table 2:** Summary of studies in periparturient dairy cows investigating the effects of supplemental essential fatty acids on liver, immune system, inflammation, and reproductive performance

Nutraceutical^a^	Form	Dose^b^	Period	Main outcomes^c^	Reference
EPA (C20:5n-3) and DHA (C22:6n-3)	Fish oil	1.5 (22 to 250 g oil/d)	− 49 to 160 DFC^6^	Lower production of TNF by neutrophils stimulated with LPS as compared with those from cows fed palm oil	[47, 48]
n-3 PUFA (C20:5n-3; C22:6n-3)				Decreased liver ketogenesis; Activates PPARG reducing the production of TNF by leukocytes and partially reversing the insulin resistance caused by this cytokine, thus increasing the glucose availability and the energy balance	[49, 50]
CLA (*cis-*9,*trans-*11 and *trans-*10,*cis*-12 C18:2n-6)	CLA	50–100 g/d	− 21 to 252 DFC	Inhibited the LPS-induced inflammatory activity in macrophages; Increased albumin and cholesterol concentrations in early lactation	[51, 52]
n-6 PUFA (C18:2 *cis*-9, *trans*-11 and *trans*-10,*cis*-12)				Increased secretion of VLDL and apolipoprotein B100, decreasing cellular accumulation of triglycerides from palmitic acid, increasing DMI, reducing NEB and lipomobilization (lower NEFA and BHB levels in blood)	[53, 54]
				Mitigation of the oxidative stress status Protection of paraoxonase against oxidative inactivation	[53, 54]
ALA (C18:3n-3)	Whole flaxseed			Higher conception rate at the first artificial insemination	[55]
				No embryo mortality with flaxseed compared to 15% with Megalac or 8% with soybean	[56–58]
	Extruded flaxseed			Higher number of small follicles compared to cows fed extruded soybeans	[59]
		4–5	− 21 to 100 DFC	Longer interval between PGF2α injection to behavioral estrous and estradiol peak. Longer duration of behavioral estrous. Fewer days from the first artificial insemination to conception and open day	[21]
	Flaxseed oil			Higher cleavage rate as compared with cows supplemented with saturated fatty acids	[60, 61]
	Encapsulated flaxseed oil	3.8 (242.2 to 700 g oil/d)	114 DFC to ovum pick up	Higher number of 2 to 7 mm follicles compared to cows fed sunflower oil or saturated fatty acids +EI: Lower concentration of estradiol and estradiol: progesterone ratio in estradiol active follicles	[31, 62]
EPA (C20:5n-3) and DHA (C22:6n-3)	Algae product containing 10% DHA	100 g/d	27 to147 DFC	Increased resumption of estrous at 58 DFC and increased pregnancy per artificial insemination (Reduced days to pregnancy by 22 d compared with control)	[63]
	Fish oil	200 to 500 g/d	− 21 to 21 DFC	Decreased pregnancy losses and larger ovulatory follicles; Higher cleavage rate as compared with cows supplemented with saturated fatty acids	[61, 64]

^a^ *ALA* Alpha-linolenic acid, *EPA* Eicosapentaenoic acid, *DHA* Docosahexaenoic acid, *TNF* tumor necrosis factor – alpha, *LPS* lipopolysaccharide, *PPARG* peroxisome proliferator-activated receptor gamma, *VLDL* very low-density lipoprotein, *DMI* dry matter intake, *NEB* negative energy balance, *NEFA* non-esterified fatty acids, *BHB* beta-hydroxybutyrate, *PGF2α* prostaglandin F2α

^b^ Expressed as % of dry matter whenever not differently indicated

^c^ Days from calving

Thus, high concentration of AA in the follicular fluid increases the production of estradiol, impairing oocytes implantation. An indirect effect driven by altered concentrations of hormones and metabolites in the follicular fluid surrounding the oocytes has also been hypothesized to concur in ameliorated EI driven by n-3 PUFA [66]. While supplementing n-3 PUFA has shown to improve the dynamics of follicular development (FD) in the ovary (Table 2), the mechanisms controlling such effects are still unknown [21]. Finally, supplementing n-3 PUFA is also known to improve embryo survival (ES) in late pregnancy (Table 2), as n-3 PUFA is essential for developing the central nervous system and reproductive system in unborn calves [56–58].

### Methyl donor supplementation

#### Choline

Choline (beta-hydroxyethyltrimethylammonium hydroxide), an essential nutrient with various functions, is an essential component of various membrane phospholipids (phosphatidylcholine, lysophosphatidylcholine, choline plasmalogen, and sphingomyelin), a precursor for the synthesis of the neurotransmitter acetylcholine, and a source of labile methyl groups [72]. Besides, it is involved in lipid metabolism as a lipotropic agent. In fact, it is also a component of very low-density lipoproteins (VLDL) that carry fatty acids from the liver to peripheral tissues, thus playing a pivotal role in preventing fatty liver syndrome. Its need is strictly related to the other components of the 1-carbon metabolism, such as methionine, betaine, folic acid, and vitamin B12. The latter represents an important set of reactions involved in the synthesis of lipids, proteins, nucleotides, antioxidants, and methylation reactions [73].

Although choline is contained in various dietary feedstuffs, such as soybean, cottonseed, sunflower, and rapeseed [74], it is extensively degraded in the rumen [75], resulting in a low intestinal absorption from dietary sources. Apart from the diet, another possible source of choline is represented by the *de novo* synthesis of phosphatidylcholine through sequential methylation of phosphatidylethanolamine with S-adenosylmethionine (SAM) as the methyl donor [72]. Methyl groups can also be synthesized *de novo* by the tetrahydrofolate (THF) system [72]. Since both are interchangeable as methyl donors, choline is considered an essential nutrient for mammals when there is a lack of methionine and folates [76]. Around calving, the endogenous synthesis does not always satisfy the cow’s needs, considering the high output of methylated compounds through milk, low input from the diet, and possible short supply of methionine. Additionally, when choline is lacking, the methyl group metabolism is conservative, with a low rate of catabolism and a high rate of *de novo* synthesis via the THF system. Consequently, choline can be seen as a limiting factor in early lactating cows [76].

It has been suggested that supplementation of rumen-protected choline (RPC) can affect performances (Table 3), but the results are not always consistent. The increase in DMI due to RPC supply may explain the effects on milk production. For instance, the function of choline as a lipotropic agent can have positive effects on butterfat production, as it improves lipid metabolism, increasing the VLDL synthesis, availability of FA for the mammary gland, and its incorporation into phospholipid membranes around fat globules. The composition of milk fatty acids can be influenced by the increased availability of preformed fatty acids via VLDL, as well as the milk protein yield, mainly because choline serves as methyl source sparing methionine [85]. The main effects of RPC supplementation are indeed on liver function (Table 4), reducing fat deposition in the liver. To a greater extent, the biosynthesis of phosphatidylcholine (PC) comes from the CDP-choline pathway (which involves choline in the first step). In turn, PC is responsible for hepatic lipoprotein assembly and secretion into bloodstream as, mainly, VLDL. Thus, choline improves VLDL synthesis through NEFA esterification into TAG, contributing to the decrease of NEFA load in the liver [95]. The latter leads to reduction of both BHB levels and fatty liver incidence, which is responsible for impaired gluconeogenesis [96]. Moreover, positive effects were detected on the immune system, where abundant antioxidant content and reduced oxidative stress status were observed in polymorphonuclear leukocytes (PMNL) and monocytes phagocytosis associated with RPC supplementation improved in the periparturient period [90].

**Table 3:** Summary of studies in periparturient dairy cows investigating the effects of supplemental methionine or choline on performances

Nutraceutical	Treatment	Period, relative to calving	Main outcome	Reference
RPC	45–200 g/d	From − 41 to 140	Higher milk yield	[77, 78]
	40–60 g/d	From − 21 to 90	Higher milk protein	[79]
	25–200 g/d	From − 41 to 63	Higher milk fat	[77]
	56–60 g/d	From − 25 to 80	Greater DMI	[80]
RPM	0.07–0.19% DM	From − 28 to 60	Higher milk yield	[81, 82]
	0.09–0.1% DM	From − 28 to 60	Higher milk protein	[81]
	0.09–0.1% DM	From − 28 to 60	Higher milk fat	[81]
	0.06–0.1% DM	From − 28 to 140	Greater DMI	[81, 83]
	0.09% DM	From − 28 to 0	Greater calf weight at birth and in first weeks	[84]

*RPC* rumen protected choline, *RPM* rumen protected methionine, *DM* and *DMI* dry matter and dry matter intake, respectively

**Table 4:** Summary of studies in periparturient dairy cows investigating the effects of supplemental methionine or choline on liver, immune system, udder, pancreas, and uterus

Nutraceutical	Treatment	Period	Tissue/Cells	Main outcome	Reference
RPC	45–75 g/d	From − 45 to 63	Liver	Reduced liver glycogen and greater plasma glucose concentration	[83, 86]
	60–75 g/d	From − 45 to 63	Liver	Increased the rate of triglycerides export with reduced liver TAG accumulation	[87]
	45–60 g/d	From − 45 to 43	Liver	Lower NEFA postpartum increase with overall decrease in plasma NEFA concentration	[88]
	45 g/d	From − 41 to 30	Liver	Higher plasma α-tocopherol	[88]
	50–100 g/d	From − 21 to 45	Liver	Increased serum haptoglobin	[89]
	50–100 g/d	From − 21 to 45	Pancreas	Increased serum insulin	[83, 89]
	60 g/d	From −25 to 80	Immune system	Lower diseases incidence	[80]
	60 g/d	From − 17 to 21	Udder	Higher colostrum IgG and better average daily gain in calves	[78]
	60 g/d	From − 21 to 30	PMNL and monocytes	Overall better redox status in PMNL and improved monocytes phagocytosis	[90, 91]
RPM	0.07–0.19% DM	From − 28 to 60 and *in vitro* culture	Immune system	Better inflammatory status, innate immune responsiveness and neutrophil function (both in calves and cows)	[92]
	0,09% DM	From − 28 to 0	Uterus	In calves, increased 1-carbon metabolism, transsulfuration pathway, and TCA cycle intermediates	[93]
	0.08–0,09% DM	From − 28 to 0	Uterus	Better calves’ adaptation to extrauterine life	[84, 94]
	0,09–0.1% DM	From − 28 to 60	Immune system	Better inflammatory and oxidative status	[92]
	0.07–0.19% DM	From − 28 to 60	Liver	Increased APP- concentrations (PON, cholesterol, albumin)	[92]
	0.07–0.19% DM	From − 28 to 0	Liver	Reduced APP- concentrations (ceruloplasmin, serum-amyloid A)	[82]
	0,09–0.1% DM	From − 28 to 60	Liver	Increased antioxidants blood concentrations (β-carotene, tocopherol, glutathione)	[92]
	0.07–0.19% DM	From − 28 to 0	Liver	Increased carnitine and glutathione synthesis	[82]

*RPC* rumen protected choline, *RPM* rumen protected methionine, *TAG* triacylglycerol, *NEFA* non-esterified fatty acids, *PMNL* polymorphonuclear leukocytes, *TCA* tricarboxylic acid cycle, *APP* negative acute phase protein, *PON* paraoxonase

#### Methionine

Methionine, a limiting amino acid in dairy cows [97] essential for milk protein synthesis, is involved in cysteine, glutathione, and taurine synthesis [98] and plays a central role in 1-carbon metabolism [73]. Considering that methionine and choline share similar fate [99], they also exhibit many common effects, such as the role in lipoprotein synthesis and as a methyl donor. In these pathways, SAM, which is synthesized from methionine, can be used as a precursor for phosphatidylcholine and homocysteine. Homocysteine, an intermediate of the 1-carbon metabolism, can enter the transsulfuration pathway, through which cysteine can be synthesized. Cysteine is a precursor of taurine and glutathione, two important antioxidants. The NRC [100] proposed a daily methionine requirement of 2.4% of metabolizable protein for lactating cows; however, diets frequently do not meet this need. Therefore, supplementation in rumen-protected form is needed because, like choline, methionine is degraded in the rumen.

Rumen-protected methionine supplementation in the first 2 weeks after calving enhances methionine serum concentrations, improving its availability, as demonstrated by Dalbach et al. [101]. Methionine can be supplemented as hydroxy-analog or in physically encapsulated form. Milk yield and butterfat can be positively affected by methionine supplementation (Table 3), even though the main effects have been observed in protein yield due to improved amino acid requirements [102]. The effects on milk yield and fat could be related to the enhanced availability of nutrients because of the positive effect on DMI. In fact, methionine supply helps in maintaining constant rates of DMI prepartum and in increasing DMI in early lactation. This result is consistent in many studies, which may be due to improved inflammatory status, reduced oxidative stress, and enhanced liver function related to methionine supplementation [81]. The improvement in these functions suggests that high-producing dairy cows adapt successfully to the new lactation and overall to the transition period following methionine supplementation.

Methionine supplementation may also have positive implications on immune cell function (Table 4) with increased phagocytosis from neutrophils, improved oxidative burst capacity, greater T-lymphocyte proliferation, and blood neutrophil-killing capacity [90, 92, 103]. Late pregnancy supply of methionine may have positive implications on calf development (Table 3) with greater calf body weight at birth and in the first weeks of life. Proper maternal supply could contribute a greater amount of available nutrients for the fetus and also improved colostrum quality, resulting in better inflammatory status and innate immune response [104].

### Live yeast and yeast-based products

There is a growing interest from livestock producers to find alternatives to antibiotics and antimicrobials for the enhancement of growth performance, general animal health, and well-being [105]. Currently, the most utilized and studied approaches are live yeast and yeast-based products derived from the strain *Saccharomyces cerevisiae*.

#### Effect of live yeast and yeast-based products on rumen activity modulation and performances

In ruminant nutrition, strains of this eukaryotic microbe help to stabilize ruminal pH and to activate fiber-degrading bacteria in the rumen, leading to improved fiber digestibility [106]. Several works have indicated a higher abundance of lactate-using bacteria (e.g. *Megasphaera* and *Selenomonas*), thus confirming the role of yeast in decreasing lactic acid concentration and helping maintain normal ruminal pH. Yeast supplementation also increased the relative abundance of fibrolytic-degrading bacteria, such as *Fibrobacter* and *Ruminococcus*, which would enhance fiber digestion in the rumen. These findings are in accordance with observations reported more recently by Uyeno et al. [107], who determined that supplementing mid-lactation dairy cows with 10 g of active yeast cells on a daily basis for 21 days activated fibrolytic bacteria in the rumen.

Regarding performance outcomes (Table 5), several studies have reported positive effects, such as increased DMI [111] and milk production [116, 117] when cows were fed *Saccharomyces cerevisiae* fermentation products, while other indicated that supplementation did not affect DMI or milk yield [118, 119]. In their meta-analysis, Poppy et al. [120] showed that supplementation with *Saccharomyces cerevisiae* fermentation products increased DMI of dairy cows in early lactation (< 70 days after calving). However, interaction effects between fermentability of the basal diet and yeast supplementation on DMI were not addressed. Recently, in a metabolic study on beef heifers, Shen et al. [121] found that supplementation of a high-grain diet (52.8% starch) with *Saccharomyces cerevisiae* fermentation products elevated the ruminal minimum pH and reduced the duration of pH < 5.6 by 6 h compared with control heifers. The study also reported improved ruminal and total-tract NDF digestibility, suggesting that the negative effects of feeding a high-starch diet can be attenuated by supplementing with yeast fermentation products. Similarly, Shi et al. [122] investigated the effects of supplementing with yeast fermentation products on milk production and DMI. The authors observed a transiently increased DMI on days 1 and 5 after calving followed by an increased feed efficiency during the post-fresh period. Overall, researchers have suggested that yeast cultures may cause a number of effects in the rumen, including increased pH, numbers of cellulolytic bacteria, and rate or extent of ruminal fiber digestion and altered VFA concentrations. On the basis of these previous results, yeast cultures may increase fiber digestion, which could increase the rate of passage and, therefore, improve DMI [111, 123].

**Table 5:** Summary of studies in periparturient dairy cows investigating the effects of supplemental live yeast or yeast-based products on performances in the prepartum and Early lactation

Nutraceutical	Treatment	Period	Main outcome	Reference
Live yeast	5 g/d/head	Early lactation (40 d in milking)	Greater VFA and rumen pH Greater fibrolytic and lactate-using bacteria	[108]
	2.5 g/d/head precalving and 10 g/d/head postcalving	From − 14 to 70 d relative to parturition	Higher rumen acetate proportion and lower ammonia nitrogen in early lactation Greater % of milk fat	[109]
Yeast culture	56 g/d/head	From calving to 14 weeks postpartum	Greater milk yield, + 1.4 kg/d + 1.6 kg/d of 3.5% FCM + 1.7 kg/d of ECM + 0.07 kg/d of milk fat	[110]
	60 g/d/head	From − 21 to 140 d relative to parturition	Higher DMI in the prepartum, + 2.10 kg/d Higher DMI in the postpartum, + 1.80 kg/d	[111]
	20 g/d/head	From − 21/− 28 to 41 d relative to parturition	Higher DMI in the first 2 d of lactation, + 5.94 kg/d + 0.14% of milk fat	[112]
Yeast culture + Enzymatically hydrolized yeast	28 g/d/head	From calving to 14 weeks postpartum	+ 1.6 milk yield, kg/d + 1.8 kg/d of 3.5% FCM + 1.9 kg/d of ECM + 0.08 kg/d of milk fat + 0.07 kg/d of milk protein	[113]
*Saccharomices cereviseae* fermentation product	16.0 ± 0.7 g/d prepartum and 18.9 ± 0.5 g/d postpartum	From 29 to 42 d relative to calving	+ 0.36% of milk fat + 0.91 of milk urea nitrogen Multiparous, higher BHB	[114]
	19 g/d/head	From 28 to 42 d relative to calving	Supplementing SCFP and reducing dietary starch content after calving may reduce inflammation and improve health status of the animal after calving	[115]
	56 or 112 g/d/head	From −4 weeks to 4 weeks relative to calving	+ 5.15 milk yeald, kg/d + 0.14 protein, kg/d + 0.26 lactose, kg/d lower SCC (− 80.5 cells/μL)	[116]

*VFA* volatile fatty acid, *FCM* fat corrected milk, *ECM* energy corrected milk, *DMI* dry matter intake, *BHB* beta-hydroxybutyrate, *SCFP* *Saccaromices cereviseae* fermentation product, *SCC* somatic cells count

Supplementation with yeast fermentation products has shown to decrease milk urea nitrogen concentration in cows fed high-starch diet. Comparatively, supplementing cows yeast fermentation products led to higher blood glucose and lower BHB concentration at 42 d after calving, suggesting a greater energy supply from the diet. Conversely, Nocek et al. [113] found an increase of milk, fat-corrected milk, and energy-corrected milk in early lactation cows supplemented with a yeast culture and yeast culture plus enzymatically hydrolyzed yeast.

#### Effect of live yeast and yeast-based products on mucosal and systemic immunity, and metabolic response

Little is known regarding the direct and indirect positive effects on the immune system and its subsequent biomarkers. Thereby, mitigation of negative effects associated with metabolic stresses and disease remains limited as well (Tables 5 and 6). Such responses could be attributed to improved energy status due to the effects on digestive function or to activation of the immune system through sensing of yeast components in the gut and subsequent cross talk between immune cells. However, the exact mechanism is unclear, especially stressing the potential of the yeast response to influence mucosal immunity.

**Table 6:** Summary of studies in periparturient dairy cows investigating the effects of supplemental live yeast or yeast-based products on rumen epithelium, liver, colon, and plasma biomarkers

Nutraceutical	Treatment	Period	Tissue/Cells	Main outcome	Reference
Live yeast	1 × 10^10^/d/head	From − 21 to 21 d relative to calving	Rumen	Dry cows responded rapidly to live yeast with Greater abundance of *TLR4* and *IL10* prepartum	[124]
			Colon	Greater abundance of *DEFB1*	
Live yeast	2.5 g/d/head precalving and 10 g/d/head postcalving	From −14 to 70 d relative to calving	Liver	Greater glycogen	[109]
			Plasma	Greater phosphorous concentration	
*Saccharomices cereviseae* fermentation product	19 g/d/head	From 28 to 42 d relative to calving	Rumen	Greater abundance of *IGFBP6* In cows fed a high-starch diet increased abundance of *BDH2*	[122]
	19 g/d/head	From − 28 to 42 d relative to calving	Plasma	Lower haptoglobin at 7 d postpartum	[115]
	19 g/d/head	From −29 to 42 d relative to calving	Liver	Tendency to increase *PCK1* mRNA abundance	[114]
			Liver	Decreased cholesterol	
			Plasma	Increased cholesterol	
	56 or 112 g/d/head	From −4 weeks to 4 weeks relative to calving	Plasma	Higher glucose between partum and + 3 d Higher urea at partum and + 1 d. With the dosage of 112 g/d/head, higher BHB at 3 d postpartum Lower cortisol	[116]

*TLR4* toll-like receptor 4, *IL10* interleukin 10, *DEFB1* β-defensin, *IGFBP6* insulin like growth factor binding protein 6, *BDH2* 3-hydroxybutyrate dehydrogenase 2, *PCK1* phosphoenolpyruvate carboxykinase 1, *BHB* beta-hydroxybutyrate

Accordingly, Chen et al. [125] detected a higher expression of *TLR* receptors in ruminal epithelium of steers classified as acidosis resistant compared with those classified as susceptible. On the other hand, mechanisms of endotoxin (LPS) tolerance are present in intestinal epithelial cells and act to avoid deleterious TLR activation by a toll-interacting protein [126]. Minuti et al. [16] concluded that the ruminal epithelium during the transition period most likely adapts to an increase in rumen LPS content due to higher diet fermentability, resulting in greater VFA production and suboptimal pH driving greater bacterial lysis. Feeding live yeast results in greater abundance of *TLR4* expression in ruminal epithelium, leading to a quicker response by innate and adaptive immunity [124]. On the other hand, further studies need to be conducted in order to highlight the mechanism behind the interaction between supplemented yeast, rumen epithelium, and innate immunity, allowing to define as positive the better degree of activation to the shorter time of resolution of a certain inflammatory response.

In response to an acute inflammatory stress induced by lipopolysaccharide, Fink et al. [127] reported that yeast products improved the health of beef cattle during the receiving period (i.e. the first 50 days in the feedlot) upon arrival to a feedlot. Duff and Galyean [128] also reported that improvements in health (i.e. decreased morbidity) may be observed in yeast-supplemented cattle exposed to stress, such as that associated with bovine respiratory disease or bovine viral diarrhea. Sanchez et al. [129] reported that yeast cell wall supplementation in receiving cattle enhanced the metabolic response to an acute immune challenge (i.e. lipopolysaccharide), thus improving the probability of recovery and enhanced efficiency of incoming cattle. Specifically, the authors [129] reported that energy metabolism and nutrient utilization may have been enhanced in yeast supplemented heifers prior to an immune challenge characterized by increased insulin, and decreased NEFA accompanied by alterations in blood urea nitrogen.

Effects of supplementing live yeast or yeast fermentation products during the transition period are inconsistent if we look at the inflammation and immune response (Table 5). However, Knoblock et al. [115] reported a marked reduction of haptoglobin 7 days after calving in cows supplemented with a *Saccharomyces cerevisiae* fermentation product, indicating that reduced inflammation was likely achieved by increased feed intake after calving. It is right to report that these kinds of commercial yeast-products contain multiple vitamins and antioxidants (i.e. polyphenols), bioactive compounds, including fermentation end products, β-glucans, and other components of the yeast cell, making difficult to discern the specific role of yeasts. Thus, it is difficult to evaluate which specific or in-combination compounds modulate the immune response reported in both human and animal studies. For example, a key constituent of yeast cell walls, β-glucan, has been shown to enhance defense against infections in rodent models [130]. β-glucans are not efficiently absorbed, but they interplay with gut-associated lymphoid tissues, which, in turn, could substantially influence circulating immune cells [106]. These interactions provide a mechanism, whereby signals at the mucosal surface (such as recognition of antigens and release of cytokines) of the gastrointestinal tract can broadly affect the function of leukocytes (macrophages, neutrophils, and lymphocytes) that migrate to damaged or infected tissues [131]. Recently, Yuan et al. [132] found that a product containing yeast culture plus enzymatically hydrolyzed yeast at the rate of 0, 30, 60, or 90 g/d from 21 d before to 42 d after calving linearly increased plasma anti-ovalbumin IgG levels following 3 ovalbumin challenges, which, in turn, indicates enhanced humoral immunity. The authors also concluded that supplementation with a product containing yeast culture plus enzymatically hydrolyzed yeast enhanced measures of humoral and mucosal immunity and modulated uterine inflammatory signals and mammary gland health in transition dairy cows. This further suggests that the immune system could be better alerted if immunogenic stimuli occur.

### Phytoproducts

Phytochemical or phytoextracts are bioactive compounds naturally present in plants and products of secondary metabolism. These have raised great interest from the scientific community from *in vitro* studies to *in vivo* application to prevent clinical conditions and improve performances. These compounds, at the chemical level, can be categorized into two major categories: carotenoids and polyphenol. Polyphenols (PP) comprise about 5000 different molecules that are sub-divided into 5 classes: phenolic acids, flavonoids, lignans, and stilbenes. Some of the more common plants containing polyphenols, notable for their curative properties, include thyme and oregano (thymol), clove (eugenol), juniper (pinene), dill (limonene), cinnamon (cinnamaldehyde), hot peppers (capsaicin), tea tree (terpinene), garlic (allicin), and anise (anethol) [133]. Other plants of interest are aloe, yerba mate, pomegrate, sylimarin, green tea, and *Hottunya cordata*. Phytochemicals have been investigated as feed additivities for their potential use as antioxidants, antimicrobiotics [134], and immune stimulators or modulators of rumen fermentation [135, 136] to improve the general welfare status (Table 7), metabolism (Table 8), and reduce antibiotic use [105]. These products can affect animals by modulating appetite or digestive functions and processes (e.g. fiber digestibility, level of volatile fatty acids production in rumen), interacting with immune, endocrine or metabolic systems, and increasing their performance (milk yield and composition, fertility) [145, 146].

**Table 7:** Summary of studies in periparturient dairy cows investigating the effects of supplemental phytoproducts on performances in the prepartum and early lactation

Nutraceutical	Treatment	Period	Main outcome	Reference
EO mix (thymol, eugenol, vanillin, guiacol, limonene	1 g/cow/d	From −21 d to 42 d relative to calving	No effect	[137]
	2 g/cow/d	28 d	DMI decrease. Small effects on digestion, ruminal fermentation, milk production and composition. Ruminal pH increase	[138]
	1.2 g/cow/d	From −4 weeks before calving to 15 weeks after	DMI decrease but milk yield was maintained	[133]
Herbal choline	17 g/cow/d (0.071% of TMR)	1 year	Improve milk yield and fertility in first lactation cows. Reduction of abortions and mastitis and respiratory problems. Hypocalcemia disorders increase	[139]
Tea saponin	20, 30, 40 g/cow/d	8 weeks treatment	Intermediate dose (20, 30 g/d) had no effect on feed intake. High dose (40 g/d) decreased DMI and milk yield)	[140]
EO mix (menthol, eugenol, anethol)	1.2 g/cow/d	20 d	Improved feed efficiency and calcium homeostasis at rumen level	[141]

*EO* essential oil, *DMI* dry matter intake

**Table 8:** Summary of studies in periparturient dairy cows investigating the effects of supplemental phytoproducts on cow metabolism

Nutraceutical	Treatment	Period	Tissue/Cells	Main outcome	Reference
EO mix (thymol, eugenol, vanillin, guiacol, limonene	1 g/cow/d	From −21 d to 42 d relative to calving	Blood	γ-glutamyltransferase increase	[137]
EO (curcum oleoresin or garlic excract or capsicum oleoresin)	2 g/cow/d	9 d treatment	Blood	Subtle or no effect on blood cells, nutrient digestibility, antioxidant status and mRNA liver enzymes expression. Immune-stimulatory effect by CD4 cells activation and expansion	[142]
EO (allicin or pinene)	5 g/cow/d *Allium sativum* standardized at 1.5% of allicin; 2 g/cow *Juniper communis* standardized at 35% pinene	21 d	Rumen	DM, OM and CP ruminal truly digestibility increase	[143]
Quebracho tannin	90 g/cow/d	6 weeks (− 3 before and + 3 after calving)	Blood	Antiketogenic effect decreasing BHB	[144]
Tea saponin	20, 30, 40 g/cow/d	8 weeks treatment		Tea saponin may reduce oxidative stress and improve immune system	[140]

*EO* essential oil, *DM and DMI* dry matter and dry matter intake, respectively, *CD4* cluster differentiation-4, *OM* organic matter, *CP* crude protein, *BHB* beta-hydroxybutyrate

Implementation these substances during the transition period of dairy cows could improve rumen functions that control ruminal pH and prevent sub-acute acidosis conditions, increasing duodenal flow of protein and reducing methane production and energy losses [147, 148].

Phenols are resistant to rumen microbial degradation and, thus, could reach the small intestine [142]. This potential could be translated into improved digestibility, microbicidal activity against pathogens, and better antioxidant status and immune response, which compensate the negative effect of negative energy balance (NEB) that characterizes the transition period. Concerning the method of administration, these compounds could be obtained from different plant parts (peel, seed, leaves or stem) and added into TMR as powder or essential oil (EO) form. The dosage of administration depends on the plant species, chemical compound, and concentration, while either a single extract or blend can be used for administration. Most phytoextracts are applied in the form of essential oils that are recognized to have antimicrobial activity. In particular, their beneficial effects occur at the bacterial membrane level by changing the structure and fluidity [149], inhibition of enzymes [150] and proteins, RNA and DNA of the cells [151], or altering the flow transmembrane cations [150]. Although both gram-positive and gram-negative bacteria are affected by EO activity [152], gram-negative are less sensitive [153–155], which could affect their potency and selectivity on rumen microbiota modulation mainly to reduce methane losses [137]. Drong et al. [137, 156] evaluated the effect of a commercial EO-mix (Tables 7 and 8) on performance, energy metabolism, and immunological parameters of cows during the transition period. The results showed no effect of the EO-blend on energy status or milk production and decreased dry matter intake, which may have been affected by the experimental design and dose administration. Benchaar et al. [138] and Tassoul et al. [133] also reported decreased dry matter intake without influencing nutrient digestibility, ruminal fermentation, and milk production using an EO-blend containing thymol, eugenol, vanillin, and limonene. Comparatively, Oh et al. [142] reported an immune-stimulatory effect due to activation and induction of the expansion of CD4 cells using an EO-mix (garlic, capsicum oleoresin) pulsed into the rumen. Braun et al. [141] highlighted the potential of EO to activate cation-transporting proteins, increasing the uptake of cations like calcium and ammonium at the ruminal epithelial level; in particular, calcium uptake plays an important role in the milk fever metabolic disorder. Yang et al. [143] suggested instead that garlic or juniper berry EO increased dry and organic matter digestibility correlated with increased crude protein rumen digestibility.

Tannins, a type of water-soluble polyphenolic compounds in plants, are considered a natural antioxidant due to the capability of their aromatic rings to combine with free radicals and form stabilized phenoxyl radicals [157]. Tannins and saponins have also demonstrated to modify the ruminal biohydrogenation process and milk fatty acid profile. Measures for using tannins must consider their antinutritional properties (that could cause dry matter intake and digestibility reduction with consequences on productive and reproductive performances, depending however by type (condensed or hydrolysable tannins) and dose [158]. Benchaar et al. [138] reported only a minor effect of *Yucca schidigera* saponins and no effect of quebracho tree tannins on milk yield and fatty acid profile. Senturk et al. [144] investigated the influence of tannins on protein metabolism and negative energy balance, which resulted in decreased BHB. Wang et al. [140] reported that tea saponins may reduce oxidative stress and improve the immunity system, but the effect depends on the dosage supplementation.

Ruminal protected choline substitute has also raised interest as a feed plant containing herbal choline conjugates due to the antimicrobial and immune stimulating effect of its contained phytoextract. During a three-year study, Gutièrrez et al. [139] revealed that herbal choline inclusion (0.071% of the diet) increased milk yield and fertility, reducing disease (mastitis, abortions) but increasing hypocalcemia disorders.

Research has also been open to *Aloe arborescens*, which contains polysaccharides and pectins and exhibits anti-inflammatory, immune stimulant, antibacterial, and antioxidant properties [105, 159, 160].

## Conclusions

Nutraceuticals provide a valuable tool in feed additives due to their host-protecting functions (antioxidant, anti-inflammatory, antimicrobial, and cell survival effects) to increase productive and reproductive performances. Thus, administration of peripartum nutraceuticals– particularly those discussed in the present review – has drawn attention to their potential health benefits and metabolic responses. The latter opens a wide perspective to further understand the site and mode of action of these compounds towards the main organs primarily involved in the homeorhetic adaptation during the transition period, such as the gastrointestinal epithelia, liver, adipose tissue, rumen, immune system, and uterus. Even more, the data support the use of supplemental nutraceuticals in the transition period to enhance the metabolic, immune, and antioxidant system response and to reduce the release of “signals” responsible for inflammation, immune dysregulation, and metabolic adaptation impairment immediately after parturition. These mechanisms should be further investigated in-depth through a system biology approach, combining performance data with -omics techniques, such as transcriptomics, proteomics, and metabolomics. *In vitro* studies are also notably encouraged, whereby researchers could target specific hypotheses and successfully answer them at least at the molecular level. However, nutraceuticals are a large class of compounds, and thus, their efficacy is affected by many factors. Several of these include the source, the technique used for production, concentration of the compound, along with the physical condition, diet, rumen pH, animal physiology, interference among compounds and nutrients, and synergistic or antagonistic effects. For these reasons, more studies should be performed to assess the efficacy and toxicity of these natural and potent compounds as well as their role in reducing the need for antibiotics.

## Data Availability

Not applicable.
